# Development of a Logic Model for a Physical Activity–Based Employee Wellness Program for Mass Transit Workers

**DOI:** 10.5888/pcd11.140124

**Published:** 2014-07-17

**Authors:** Bhibha M. Das, Steven J. Petruzzello, Katherine E. Ryan

**Affiliations:** Author Affiliations: Steven J. Petruzzello, Katherine E. Ryan, University of Illinois, Urbana-Champaign, Illinois. Work was completed at the University of Illinois, Urbana-Champaign.

## Abstract

Transportation workers, who constitute a large sector of the workforce, have worksite factors that harm their health. Worksite wellness programs must target this at-risk population. Although physical activity is often a component of worksite wellness logic models, we consider it the cornerstone for improving the health of mass transit employees. Program theory was based on in-person interviews and focus groups of employees. We identified 4 short-term outcome categories, which provided a chain of responses based on the program activities that should lead to the desired end results. This logic model may have significant public health impact, because it can serve as a framework for other US mass transit districts and worksite populations that face similar barriers to wellness, including truck drivers, railroad employees, and pilots. The objective of this article is to discuss the development of a logic model for a physical activity–based mass-transit employee wellness program by describing the target population, program theory, the components of the logic model, and the process of its development.

## Background

Although the benefits of physical activity (PA) are widely touted, 52% of Americans do not meet PA recommendations (1). Because most American adults spend a significant amount of their day in the workplace, worksite-based PA programs have potential to promote PA in an otherwise sedentary population (2). Worksite wellness programs (WWPs) reduce health risks, increase productivity, and improve quality of life (3,4). Although WWPs are beneficial for employers and employees, many are terminated because of lack of resources, staff, and knowledge (5). Logic models help practitioners understand best practices for allocating resources and staffing to create sustainable WWPs. Logic models are a visual representation of program resources, activities, and short- and long-term outcomes. Although other logic models focusing on worksite wellness have used PA as one component of their initiative, this logic model uses PA as the centerpiece for improving mass transit employees’ health.

**Mass transit employees.** The nearly 400,000 employees of the transportation industry (6) are at increased risk of obesity, physical inactivity, and poor nutrition compared with employees of other industries because of long work hours, work shift schedules, and lack of scheduled breaks or meals (7–9). This sector lacks readily available healthy food choices and PA options, both on transportation routes and in transportation centers, and has high levels of stress and fatigue (7–9). Bus drivers have high rates of illness, death, and absence due to illness (8). Many factors influence transportation workers’ obesity prevalence and risk of excess weight gain, including individual behaviors and work environment characteristics (10). Sixty-two percent of metropolitan mass transit employees found it difficult to be physically active while at work (11). Obese drivers were less likely to engage in moderate PA and more likely to engage in sedentary behaviors compared with overweight and normal-weight drivers (11). PA-focused WWPs are important for this population. Program theories and logic models can be used by worksite wellness practitioners to allocate resources, develop activities, and evaluate outcomes for mass transit employees.

**Program theory and logic models.** Program theory is the set of assumptions about how a program should work, describing the chain of interventions and participants’ responses leading to the program’s outcomes (12). Knowledge of these assumptions helps explain why some programs work while others fail (13). Based on program theory, logic models are visual representations of the components and sequence of events needed to obtain meaningful program results, linking short- and long-term outcomes with programmatic activities, inputs, processes, outcomes, and theoretical assumptions and principles (13). Developing a logic model can enhance a program’s direction by defining program strategies (14).

Using logic models creates a sense of community and identity among program staff, participants, and stakeholders (15) by depicting the connections between a program’s resources, activities, outputs, and outcomes (16). Evaluators use logic models to examine a program’s process (17), assess overarching impact (17), and interpret evaluation findings (18). For public health dissemination, logic models provide practitioners with process and outcome data, allowing for better results (19). For a WWP, development of a logic model provides program managers, users, and evaluators with a roadmap of the program, including the intended short- and long-term outcomes (14–16). From an evaluation perspective, logic models for WWPs provide program evaluators with specific process and outcome measures for assessment.

### Logic models in PA interventions

Other logic models for WWPs have used PA as one component of the initiative, yet none has used PA as the centerpiece for improving employees’ health. The Healthier Worksite Initiative mentions an increase in PA as a long-term outcome (20). The Australian WorkHealth program used PA as a program component but not as the cornerstone (21). These models lack evidence of how program theory would be different if PA were a major resource and/or activity. Although researchers have studied using logic models for evaluating community-based PA interventions (22), to our knowledge none have studied WWPs with PA as the cornerstone.

**Logic model development.** This intervention is based on an activities-approach logic model; it connects the different planned activities together to map the process of program implementation (15). This approach explains the program’s goals, activities, and resources needed. An activities-approach model is used in program monitoring and management because it identifies steps needed to implement the program effectively and efficiently. An activities-approach logic model explains to stakeholders how program activities accomplish program goals. The logic model takes a socioecological perspective, intending to address several layers of the model, including intrapersonal, interpersonal, institutional, community, and public policy factors (23). For this study, the logic model’s components were situation, resources, activities, outputs, and outcomes.

## Creating the Logic Model

Three focus groups (n = 9, n = 11, and n = 11 participants) were conducted to determine the needs for a WWP for mass transit employees. The focus groups consisted of bus drivers (n = 16), maintenance staff (n = 6), and clerical staff (n = 9). In-depth interviews were conducted with 10 employees, 3 from each job category and the Fitness Committee chairperson. Employees were recruited via employee e-newsletters, flyers, posters, and word of mouth. A qualitative researcher with experience in developing logic models facilitated the focus groups and interviews.

Participants had varied reasons for their involvement. One of the primary reasons for participation was to learn how their employment had damaged their health and how to reverse these effects. One individual stated (Focus group 2, bus driver, participant 2), “I’ve been with [company] for 12 years now and there’s been a real physical decline in my health the longer I’ve been here.” Additionally, participants became involved at the urging of someone else, usually a family member, friend, or colleague. Employees participated to learn more about how a physical activity-based wellness model could reduce their levels of stress and fatigue and improve their weight management.

Focus group and interview questions were tailored to this population on the basis of worksite wellness literature. Participants worked at least 20 hours per week and were aged 18 or older. Content analysis was conducted to determine major themes, which informed the logic model. The model serves as a guide for the conceptual relationships between resources, activities, and outcomes. The underlying logic is that if all resources are provided, with activities carefully specified, the program will be implemented as intended, and mass transit employees will attain a series of short- and long-term positive outcomes.

## The Logic Model

A logic model (Figure) was developed to assist a mass transit district (MTD) administration and its employees with further WWP development and implementation. The MTD’s program theory development was influenced by evaluation theories and participants’ perspectives. The MTD serves a midsized, Midwestern college community. It has approximately 300 employees and supports an onsite fitness facility. Employees are categorized as operators, maintenance, and office staff. Operators drive the bus routes and are often the public face of the MTD; maintenance staff is responsible for upkeep and preservation of buses and equipment; office staff manages the day-to-day administrative operations.

The logic model assumes that, as a vital component of the community, the MTD requires a healthy, productive workforce. The Worksite Wellness Initiative at MTD supports MTD employees who are interested in becoming and remaining physically active and improving physical and mental health outcomes. The Worksite Wellness Initiative promotes PA through self-monitoring (eg, recording minutes of PA) and rewards. All MTD employees, including retirees, are eligible to participate.

**Figure:**
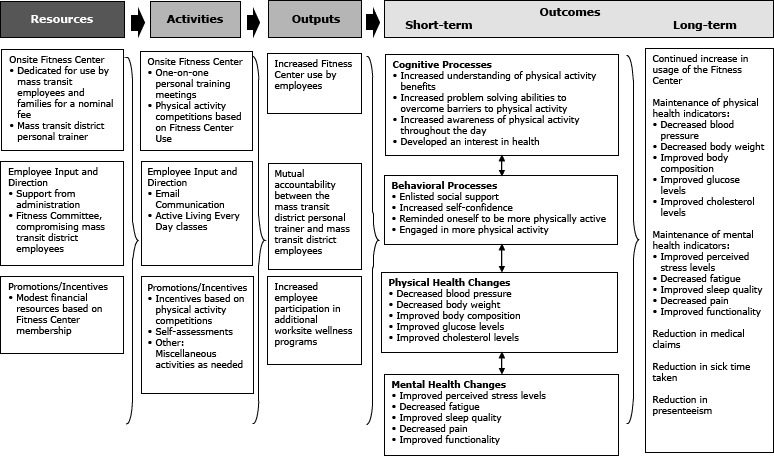
Creating a Healthier, More Productive Workforce Through Worksite Physical Activity: the logic model for a physical activity-based worksite wellness initiative for mass transit district employees.

**Table:** 

The logic model consists of a series of boxes arranged in 4 columns: Resources, Activities, Outputs, and Outcomes. The Outcomes column is further divided into Short-term and Long-term. Arrows indicate that Resources lead to activities and activities lead to outputs. A final arrow points from Outputs to Outcomes.
The Resources column consists of 3 boxes: Onsite Fitness Center, Employee Input and Direction, and Promotions/Incentives. These 3 boxes describe the resources in these categories. The Onsite Fitness Center is described as dedicated for use by mass transit employees and families for a nominal fee and has a mass transit district personal trainer. The Employee Input and Direction category has support from administration and a Fitness Committee, comprising mass transit district employees. Promotions/Incentives are modest financial resources based on Fitness Center membership.
The Activities column has 3 boxes that describe the activities in these categories: Onsite Fitness Center, Employee Input and Direction, and Promotion/Incentives. Components of the Onsite Fitness Center are one-on-one personal training meetings and physical activity competitions based on Fitness Center use. Components of the Employee Input and Direction category are e-mail communication and Active Living Every Day classes. Finally, Promotions/Incentives are based on physical activity competitions, self-assessments, and other activities.
The Outputs column has 3 boxes: increased Fitness Center use by employees, mutual accountability between the mass transit district personal trainer and mass transit district employees, and increased participation of employees in additional worksite wellness programs.
The Outcomes column has 2 subcategories: short-term and long-term. The short-term outcomes are cognitive processes, behavioral processes, physical health changes, and mental health changes. Cognitive processes have 4 short-term outcomes: increased understanding of physical activity benefits, increased problem-solving abilities to overcome barriers to physical activity, increased awareness of physical activity throughout the day, and developed an interest in health. Behavioral Processes has 4 outcomes: enlisted social support, increased self-confidence, reminded oneself to be more physically active, and engaged in more physical activity. Physical Health Changes has 5 short-term outcomes: decreased blood pressure, decreased body weight, improved body composition, improved glucose levels, and improved cholesterol levels. Finally, Mental Health Changes has 5 short-term outcomes: improved perceived stress levels, decreased fatigue, improved sleep quality, decreased pain, and improved functionality.
The 6 long-term outcomes are continued increase in usage of the Fitness Center, maintenance of physical health indicators, maintenance of mental health indicators, reduction in medical claims, reduction in sick time taken, and reduction in presenteeism.
The logic model’s key attribute is using physical activity as the cornerstone of resources. If all resources are provided, then activities will be implemented, resulting in a series of outputs and short- and long-term outcomes. The overarching goal is to create a healthier workforce.

### Resources

Resources include a dedicated Fitness Center for use by MTD employees and families for a nominal fee, a personal trainer, modest financial resources based on Fitness Center membership, support from MTD administration, and a fitness committee of MTD employees. The Fitness Center, created and maintained by MTD employees, serves as one of the primary program inputs for the Wellness Initiative Program. The Fitness Center is for the exclusive use of MTD employees and families for a nominal fee of $5 per month. Although a Fitness Center exists, many employees are hesitant to use the facility. One employee (Focus group 2, bus driver, participant 4) stated, “As a newer driver, I’m not as familiar with the other drivers so there’s not that friendship at the Fitness Center.” Other operators felt the Fitness Center was not easily accessible. One operator stated (Focus group 2, bus driver, participant 1), “The Fitness Center is here, and I don’t always make it over here. [On] this [quarter’s work schedule], I pick up all my routes either at [remote terminal] or [a shopping center], never at the actual garage.” Other drivers agreed that the fitness center location is inconvenient and would prefer to have pieces of exercise equipment at satellite locations.

Others believe the Fitness Center is not a welcoming environment. One individual (Focus group 2, bus driver, participant 4) stated, “The Fitness Center tries to act like a support center, but it isn’t always like that. I know there’s that tutorial you can use, but it’s not the same thing as having someone there to show you how to do it. I’ve met with the personal trainer a few times but he can’t always meet with me when I want, you know.” This statement is substantiated by another employee (Focus group 3, maintenance, participant 1) who reported, “I try to encourage the newbies in the Fitness Center, but not everyone is like that. Sometimes you’ll have people hazing you. When I first started, I wasn’t lifting as much as some people thought I should for a big guy, but I didn’t have the strength, either. I got some hazing for that.” Others did not feel comfortable exercising in the Fitness Center because often administrators would be exercising at the same time. Some employees believe that the MTD uses the Fitness Center to make additional money and to keep tabs on employees.

Another program input includes the MTD personal trainer. Participants report using the personal trainer to develop workouts. One individual stated, “I met with the personal trainer to develop a plan for me. It’s 3 times a week, for 45 minutes, but I can do it before I have to report for my shift.” Modest financial resources from Fitness Center memberships are used for Fitness Center maintenance and to provide additional wellness programs, such as health education programs. Financial resources from membership fees provide incentives for PA competitions, based on Fitness Center use. For example, incentives are given to members who log in the most PA sessions during a 3-month period. The MTD administration encourages employees and their families to use the Fitness Center. Finally, the Fitness Committee, made up of MTD employees, is responsible for all Fitness Center programming and maintenance.

### Activities

For the MTD, various program activities comprise the intervention. These activities include e-mail communication from the Fitness Committee to MTD employees; one-on-one personal training sessions; PA competitions based on Fitness Center use; physiological and anthropometric self-assessments; an evidence-based behavior modification class called Active Living Every Day (24); and other activities as needed. The PA competitions, based on Fitness Center usage, are one of the most important program activities. Employees earn points, tallied every 3 months, for every 30 minutes of PA completed in the Fitness Center. On the basis of points earned, participants can win gift certificates. These competitions provide incentives for employees to use the Fitness Center. One employee (Focus group 1, maintenance, participant 4) stated, “My wife and I were doing the Fitness Center challenges but we started slacking off when we started getting busier. We’re going to start back, though, because we sure do miss those gift certificates.” Another activity is one-on-one personal training meetings. Employees find one-on-one personal training meetings beneficial because they help them learn more about the Fitness Center equipment and provide an opportunity to learn about fitness markers, such as body composition, blood pressure, and weight. One-on-one personal training meetings provide employees with the opportunity to develop a meaningful and relevant PA plan, which may lead to adherence to the plan and success at becoming and remaining physically active.

For this workforce, self-assessments were important to the development of the logic model and the WWP theory. Participants assess the pros and cons of PA and evaluate their willingness to be physically active, among other factors, including their confidence to engage in PA. E-mail communication and the intra-company website are crucial components of the Worksite Wellness Initiative. These media provide an avenue of open, honest communication between the Fitness Committee and MTD employees. Use of the intra-company website provides a quick, efficient way to communicate with employees about information regarding healthy living habits, sign-ups for exercise equipment use, and schedules for health education classes. Active Living Every Day allows employees to learn more about the benefits and effects of PA in a nonthreatening environment unlike the Fitness Center, which is seen as competitive. Active Living Every Day participants feel that meeting in a classroom or break room allows them to focus on their health and well-being rather than being the fastest or strongest, as they may feel in the Fitness Center. Finally, other activities (eg, recruitment events) occur as needed.

### Outputs and outcomes

Outputs consist of increased Fitness Center usage, increased participation of employees in additional WWPs, and mutual accountability between the personal trainer and employees. The program’s resources, coupled with its activities, will lead more employees to become aware of the Worksite Wellness Initiative, which may lead to increased Fitness Center usage and participation in other WWPs. Another critical output is the mutual accountability between the personal trainer and employees. Mutual accountability builds rapport and social support, critical components to achieving and maintaining a physically active lifestyle.

Interim outcomes, which should eventually lead to the desired end results fall into 4 categories: changes in cognitive processes, behavioral processes, physical health, and mental health. For cognitive processes, participants report developing an interest in health, increasing understanding of PA benefits, developing better problem-solving abilities to overcome barriers to PA, and increasing awareness of PA throughout the day. For the behavioral processes, participants learn to enlist social support, an essential component to encouraging and maintaining PA (25). One participant reported, “Social support is really important to me. I wouldn’t go on walks or do the Fitness Center if I didn’t have someone encouraging me.” Participants adjust their behaviors by reminding themselves to be more physically active, engaging in more PA, and rewarding themselves for being more active. Participants report increased self-confidence and PA. Physical health changes are reflected in self-reported improvements in blood pressure, body weight, body composition, blood glucose, and cholesterol. Mental health changes are seen in reductions of perceived stress levels, fatigue, and pain, and improved sleep quality and functionality. Long-term outcomes include continued increase in usage of the Fitness Center and maintenance of improved physical and mental health indicators. MTD employees have reductions in medical claims, sick time taken, and presenteeism.

Several components contribute to the Worksite Wellness Initiative’s desired end results. First, the activities would change interim outcomes to create a continued increase in Fitness Center usage. Desired end results include maintenance of physical and mental health improvements for MTD employees. Together, these should result in the creation of a healthier, more productive MTD.

## Conclusions

Logic models provide practitioners with information to evaluate what they are doing (19). Based on stakeholders’ perspectives, a logic model framework is presented to illustrate the potential of the effects of a WWP on mass transit employees’ mental and physical health. To our knowledge, this article is the first to present a comprehensive logic model examining the effects of a WWP with PA as its cornerstone on mass transit employees’ health status; previous studies in this population focused primarily on social and environmental factors. This logic model has a socioecological perspective and addresses multiple levels of the issue: intrapersonal, interpersonal, institutional, and environmental factors. Intrapersonal issues include changing PA knowledge, and interpersonal processes include improving social support. Institutional factors are financial and administrative support. Environmental factors consist of a Fitness Center and Wellness Committee.

Because high rates of physical inactivity harm the workforce, a PA-based worksite wellness logic model is needed to promote worksite-based wellness. This PA-based logic model can serve as a framework for the numerous mass transit districts domestically and internationally. Although this framework was developed specifically for mass transit employees, it could be used for other populations that face similar barriers to PA due to job-related factors (eg, long work hours and work shift schedules). These populations may include truck drivers, railroad employees, pilots, and taxi drivers, all of whom have jobs requiring them to be sedentary and who may not have a feasible “active” option. Additionally, people in these jobs may have high levels of stress and issues with fatigue, which may damage their mental and physical health.

Dissemination of logic models is critical for meaningful public health impact. This model depends on having a strong PA foundation and managerial support. We believe that publication of this logic model will generate dialogue and interest among worksite health promotion professionals, especially those working in mass transit districts. Mass transit administrators and employees may also help to disseminate this logic model into other mass transit districts. We anticipate this logic model will serve as a framework for mass transit employee wellness program development and evaluation, and we intend to use this logic model framework in future research and programmatic activities involving mass transit employees, in addition to railroad employees.
